# Antitumor Activity and Mechanism of Action of the Cyclopenta[*b*]benzofuran, Silvestrol

**DOI:** 10.1371/journal.pone.0005223

**Published:** 2009-04-29

**Authors:** Regina Cencic, Marilyn Carrier, Gabriela Galicia-Vázquez, Marie-Eve Bordeleau, Rami Sukarieh, Annie Bourdeau, Brigitte Brem, Jose G. Teodoro, Harald Greger, Michel L. Tremblay, John A. Porco, Jerry Pelletier

**Affiliations:** 1 Department of Biochemistry, McGill University, Montreal, Quebec, Canada; 2 Sunnybrook Health Sciences Centre and the Department of Immunology, University of Toronto, Toronto, Ontario, Canada; 3 Goodman Cancer Center, McGill University, Montreal, Quebec, Canada; 4 Comparative Phytochemistry Department, Institute of Botany, University of Vienna, Vienna, Austria; 5 Department of Chemistry, Center for Chemical Methodology and Library Development, Boston University, Boston, Massachusetts, United States of America; Victor Chang Cardiac Research Institute (VCCRI), Australia

## Abstract

**Background:**

Flavaglines are a family of natural products from the genus *Aglaia* that exhibit anti-cancer activity *in vitro* and *in vivo* and inhibit translation initiation. They have been shown to modulate the activity of eIF4A, the DEAD-box RNA helicase subunit of the eukaryotic initiation factor (eIF) 4F complex, a complex that stimulates ribosome recruitment during translation initiation. One flavagline, silvestrol, is capable of modulating chemosensitivity in a mechanism-based mouse model.

**Methodology/Principal Findings:**

Among a number of flavagline family members tested herein, we find that silvestrol is the more potent translation inhibitor among these. We find that silvestrol impairs the ribosome recruitment step of translation initiation by affecting the composition of the eukaryotic initiation factor (eIF) 4F complex. We show that silvestrol exhibits significant anticancer activity in human breast and prostate cancer xenograft models, and that this is associated with increased apoptosis, decreased proliferation, and inhibition of angiogenesis. We demonstrate that targeting translation by silvestrol results in preferential inhibition of weakly initiating mRNAs.

**Conclusions/Significance:**

Our results indicate that silvestrol is a potent anti-cancer compound *in vivo* that exerts its activity by affecting survival pathways as well as angiogenesis. We propose that silvestrol mediates its effects by preferentially inhibiting translation of malignancy-related mRNAs. Silvestrol appears to be well tolerated in animals.

## Introduction

Cyclopenta[*b*]benzofuran flavaglines are inhibitors of translation initiation isolated from Asian plants of the genus *Aglaia* of the family Meliacae [1]–[4]. These compounds show *in vitro* activity against tumor cell lines [1], [2], promising activity in xenograft cancer models [2], [5], [6] and appear to block G2/M cell cycle progression [7]. We have previously shown that the flavagline silvestrol can re-sensitize tumor cells to standard-of-care agents, such as doxorubicin, in the Eμ-myc lymphoma model [4]. Silvestrol inhibits translation initiation by targeting the RNA helicase, eukaryotic initiation factor (eIF) 4A, and prevents ribosome loading onto mRNA templates [4].

Translation initiation is regulated by eIF4F at the level of the ribosome recruitment step. eIF4F is composed of three subunits: eIF4E, which binds to the cap structure present at the 5′ end of mRNAs; eIF4A, a DEAD-box RNA helicase implicated in preparing a ribosome landing pad for 43S pre-initiation complexes (40S ribosomal subunit and associated factors) by unwinding 5′ mRNA structure; and eIF4G, a large scaffolding protein involved in recruiting the 43S pre-initiation complex via its interaction with 40S-associated eIF3 [8]. eIF4A is an abundant translation factor that exists in a free form (referred to herein as eIF4A_f_) and as a subunit of the heterotrimeric eIF4F complex (eIF4A_c_) [9], [10]. The helicase activity of eIF4A_c_ is ∼20-fold more efficient than eIF4A_f_ and during initiation, eIF4A_f_ likely cycles through the eIF4F complex [11]. Silvestrol acts as a chemical inducer of dimerization (CID) to force an engagement between eIF4A_f_ and RNA, although how this inhibits translation initiation is not known [4].

Levels of cellular eIF4F are regulated by the target of rapamycin, mTOR [12], [13]. The extent to which translation of specific mRNAs is altered in response to changes in mTOR activity and eIF4F levels varies substantially among different transcripts and is largely dependent upon sequence elements within each mRNA, such as the presence of discrete hairpin structures in the 5′ untranslated regions [14]. Many cellular mRNAs are characterized by relatively short, unstructured 5′ UTRs (e.g. β-actin, GAPDH) that require a minimal amount of eIF4F for 43S pre-initiation complexes recruitment. These mRNAs are efficiently translated when eIF4F activity is limiting. By comparison, a select group of mRNAs is extremely sensitive to, and dependent upon, eIF4F for translation. These mRNAs typically harbor lengthy, G+C rich, highly-structured 5′ UTRs that encumber efficient RNA unwinding by the eIF4F complex and subsequently prevent efficient ribosome loading [14]. Hence, altering levels with flavaglines can exert profound gene specific effects.

The deregulation of the PI3k/Akt/mTOR signaling axis in human cancers, the finding that ectopic expression of eIF4E is oncogenic [15], [16], and the demonstration that targeted down-regulation of eIF4E displays therapeutic benefit in xenograft mouse models [17] suggest that the process of translation initiation is a potential anti-cancer target. Herein, we report that silvestrol is effective against two human xenograft models as a single agent. We provide further insight into the mechanism of action of silvestrol and show that it depletes the eIF4F complex of eIF4A and this is associated with a preferential reduction in the translation of mRNAs with structured 5′untranslated regions. Our results are consistent with the idea that silvestrol's anticancer activity is linked to its ability to preferentially block translation of highly structured, malignancy-related mRNAs.

## Results

### Structure-activity relationships of flavaglines

**Figure 1:**
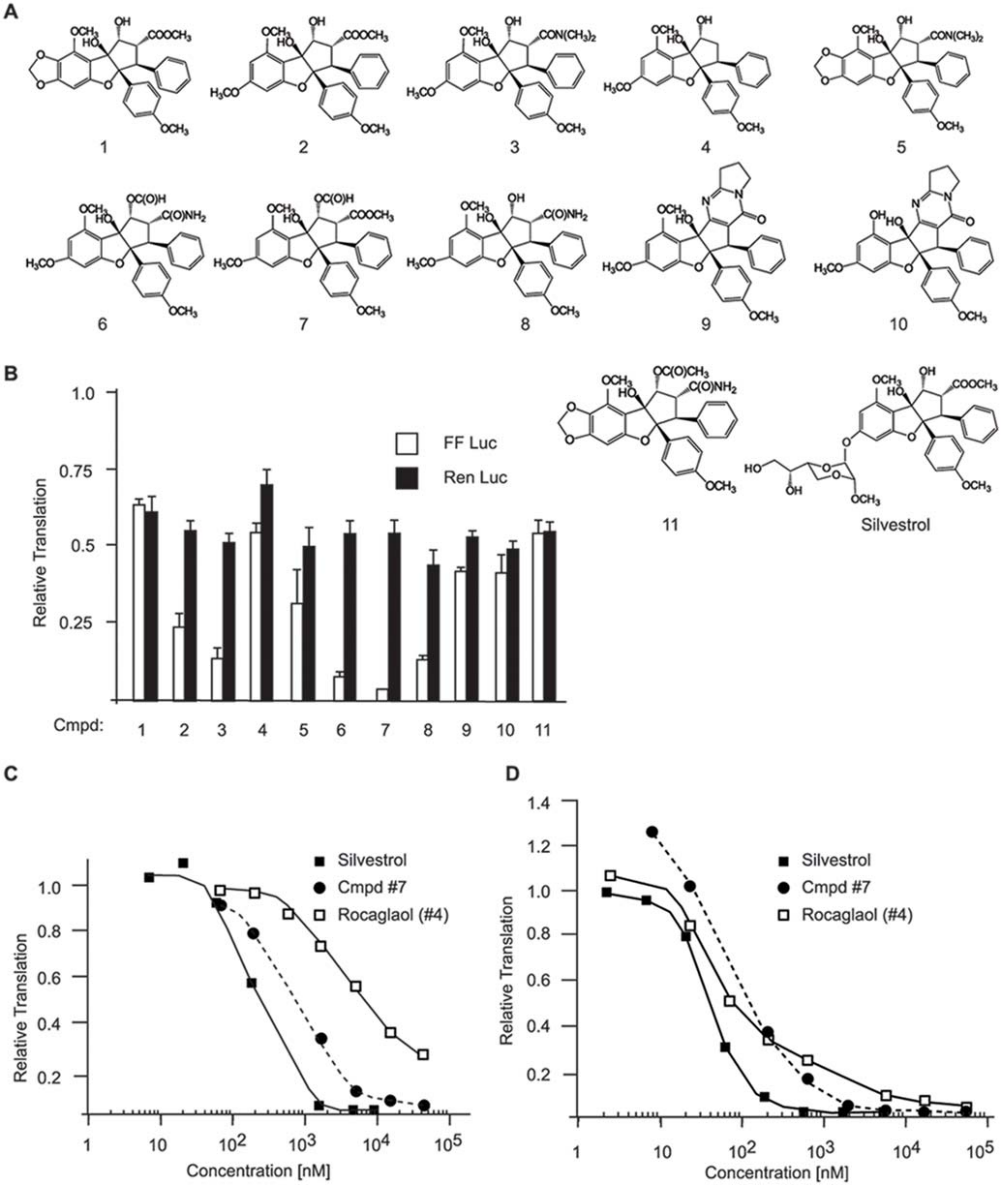
Structure-activity relationship analysis of cyclopenta[*b*]benzofurans. A. Chemical structure of cyclopenta[*b*]benzofurans tested in this study. B. Effect of cyclopenta[*b*]benzofurans on cap- and HCV-mediated translation initiation. Krebs-2 translation extracts were programmed with FF/HCV/Ren mRNA and vehicle (MeOH) or 50 µM compound. The relative activity (compared to DMSO controls) from 3 independent translation reactions is presented along with the standard error of the mean. [The partial inhibition of HCV-mediated translation has been previously documented [4].] C. Dose–dependent inhibition of translation by flavaglines in RRL extracts. Extracts were programmed with FF/HCV/Ren mRNA and firefly luciferase values determined and set relative to translations containing vehicle alone. The values obtained are the average of 2 experiments. D. Dose–dependent inhibition of translation by flavaglines in MDA-MB-231 cells. Compounds were added to cells in culture at the indicated dose for 1 h. ^35^S-methionine was added to cells 15 min before harvesting, after which TCA precipitable counts were determined and standardized against total protein content. Values are set relative to those obtained from vehicle-treated cells. The values obtained are the average of 2 experiments.

We have previously characterized silvestrol as an inhibitor of translation initiation *in vitro* and *in vivo* [4]. To determine how extensive this property is among flavaglines, we tested members of the cyclopenta[*b*]benzofurans, cyclopent[*bc*]benzopyrans, and benzo[*b*]oxepines family for their potential to inhibit protein synthesis. (Fig. 1 and Figs. S1 and S2). Among the compounds tested were rocaglaol (Fig. 1A, compound #4) [18], that has been shown to induce apoptosis and cell cycle arrest in LNCaP cells [7]; aglafolinformylester (compound #7) [19], which has been shown to inhibit cap specific translation initiation (4) and silvestrol, which can sensitize lymphomas to the cytotoxic action of doxorubicin (4). *In vitro* translation reactions in extracts programmed with the bicistronic mRNA, FF/HCV/Ren (Fig. S1A), revealed that although compound #7 inhibited FF translation to the greatest extent when compared to compounds 1–11 (Fig. 1B and Fig. S1B), it was not as potent as silvestrol *in vitro* (Figs. 1C) or *in vivo* (Fig. 1D). The closely related cyclopenta[*bc*]benzopyrans and benzo[*b*]oxepines flavagline-type compounds did not show significant activity as inhibitors of translation (Fig. S2). We focused the remainder of our studies on silvestrol since it displayed the most potent inhibition of protein synthesis among the flavaglines that we have identified to date.

### Mechanism of Action of Silvestrol

**Figure 2:**
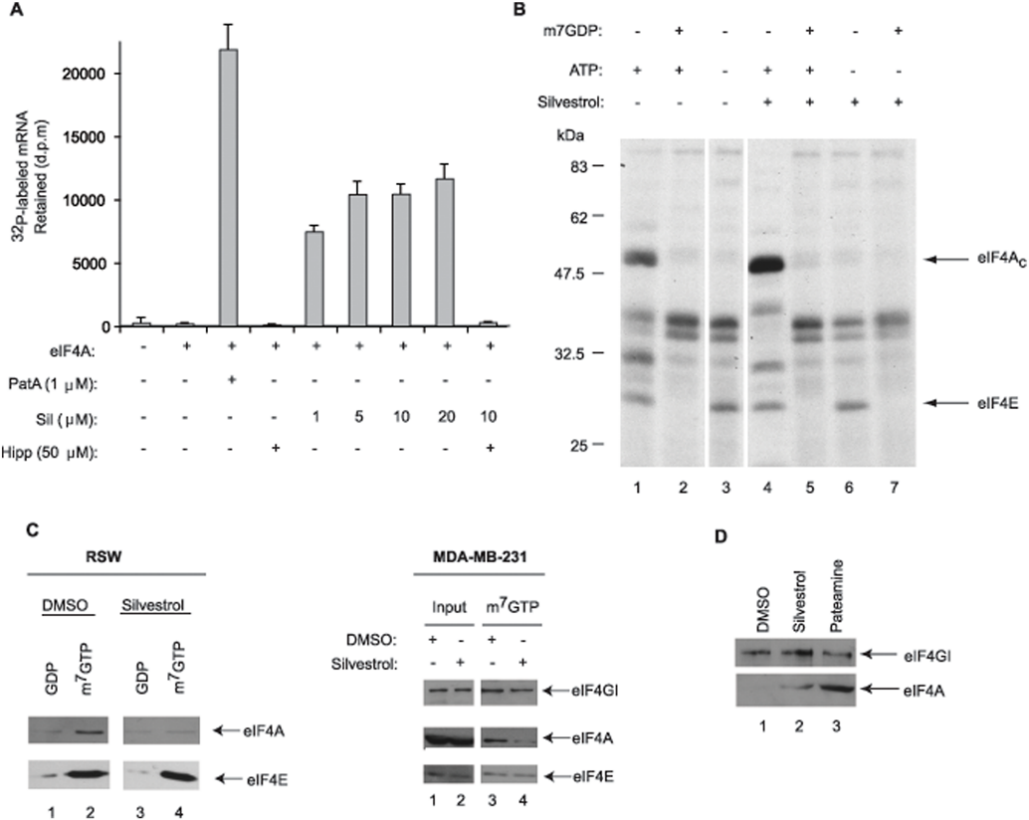
Silvestrol stimulates binding of eIF4A to mRNA. A. Silvestrol stimulates retention of eIF4AI_f_ to mRNA. Radiolabeled CAT mRNA was incubated with eIF4AI_f_ and ATP in the presence of vehicle (DMSO), pateamine A (PatA), hippuristanol [45], or silvestrol (Sil) for 2 min after which time the reactions were applied to nitrocellulose filters, washed and dried. The amount of radiolabeled mRNA retained on the filters was determined by scintillation counting. The results are the average of three experiments with the standard error of the mean shown. B. Silvestrol stimulates cap-dependent crosslinking of eIF4A_c_. Chemical crosslinking of initiation factors to oxidized ^32^P-cap labeled CAT mRNA. The presence of 20 µM silvestrol is indicated above the panel. The gel was dried and exposed to X-ray film (Kodak) at −80°C with an intensifying screen. C. Left Panel: Silvestrol reduces the amount of eIF4A_c_ in the eIF4F complex. RSW was incubated with vehicle (0.5% DMSO) or 50 µM silvestrol for 1 h followed by m^7^GTP-affinity purification. Reactions were resolved on a 10% SDS-polyacrylamide gel followed by Western blot analysis. Right Panel: MDA-MB-231 cells were treated with 25 nM silvestrol for 4 h, after which time, cell extracts were prepared and m^7^GTP-affinity purifications performed. Reactions were resolved on a 10% SDS-polyacrylamide gel followed by Western blot analysis. D. Krebs-2 extracts were treated with 50 µM of silvestrol for 10 min at 30°C. After incubation, poly (rG)-affinity purifications were performed. Eluates were resolved on a 10% SDS polyacrylamide gel and analyzed by Western blotting.

We have previously demonstrated that silvestrol acts as a chemical inducer of dimerization – promoting the interaction between eIF4A and RNA [4]. We confirmed that silvestrol increases the RNA binding properties of eIF4A using nitrocellulose binding assays (Fig. 2A). In this assay, ^32^P-labeled RNA is retained on nitrocellulose filters only when bound to proteins. Very little RNA was retained by nitrocellulose when RNA was incubated only in the presence of eIF4A (Fig. 2A, compare lane 2 to 1). In the presence of pateamine, a CID that induces eIF4A_f_-RNA interaction, a significant proportion of ^32^P-labeled RNA was retained (Fig. 2A, compare lane 3 to 2) [20]. Addition of silvestrol to the eIF4A-RNA binding reactions increased the retention of ^32^P-labeled RNA on nitrocellulose (compare lanes 5–8 to 2); an event that was inhibited by hippuristanol (compare lane 9 to 7) – a compound that binds to the C-terminal domain of eIF4A to inhibit RNA binding [21]. These results indicate that silvestrol enhances binding between eIF4A_f_ and RNA.

We have previously shown that RNA binding of eIF4A_c_ is also increased by silvestrol [4], and we now demonstrated that this was a cap-dependent phenomenon (Fig. 2B). Crosslinking of eIF4E and eIF4A_c_ from RSW to ^32^P-labeled mRNA cap structures was inhibited by the presence of m^7^GDP (Fig. 2B, compare lane 2 to 1). As previously documented [22], crosslinking of eIF4A, but not eIF4E, was ATP-dependent (Fig 2B, compare lane 3 to 1). The presence of silvestrol in the reaction stimulated the crosslinking of eIF4A_c_, but not eIF4E (compare lane 4 to 1). Crosslinking of eIF4A_c_ in the presence of silvestrol was inhibited by m^7^GDP and required ATP (compare lanes 5–7 to 4).

One consequence of increased RNA binding of eIF4A in the presence of silvestrol is that the eIF4F complex could become depleted of eIF4A_c_ – thus reducing cap-dependent translation. We therefore tested this possibility by purifying eIF4E from RSW containing silvestrol (Fig. 2C; left panel) and from cell extracts that had been prepared from MDA-MB-231 silvestrol-treated cells (Fig. 2C; right panel) using m^7^GTP-Sepharose affinity chromatography. We then probed for the presence of eIF4E and co-purifying eIF4A in m^7^GTP eluates. In both cases, the levels of eIF4A_c_ in the eIF4F complex were reduced (Figs. 2C).

To determine if components of the eIF4F complex (i.e.-eIF4G) co-sequestered with eIF4A to RNA in the presence of silvestrol, we performed pull-down assays with poly(rG)-agarose (Fig. 2D). Increased eIF4A levels were retained on poly(rG)-agarose in the presence of silvestrol and pateamine, however, this did not result in a concomitant increase of eIF4G associating with poly(rG)-agarose (Fig. 2D, compare lanes 2 and 3 to 1). [The small amount of eIF4G binding to poly(rG)-agarose may be due to this proteins' intrinsic RNA binding.]

### Inhibition of translation by silvestrol leads to mRNA discrimination

**Figure 3:**
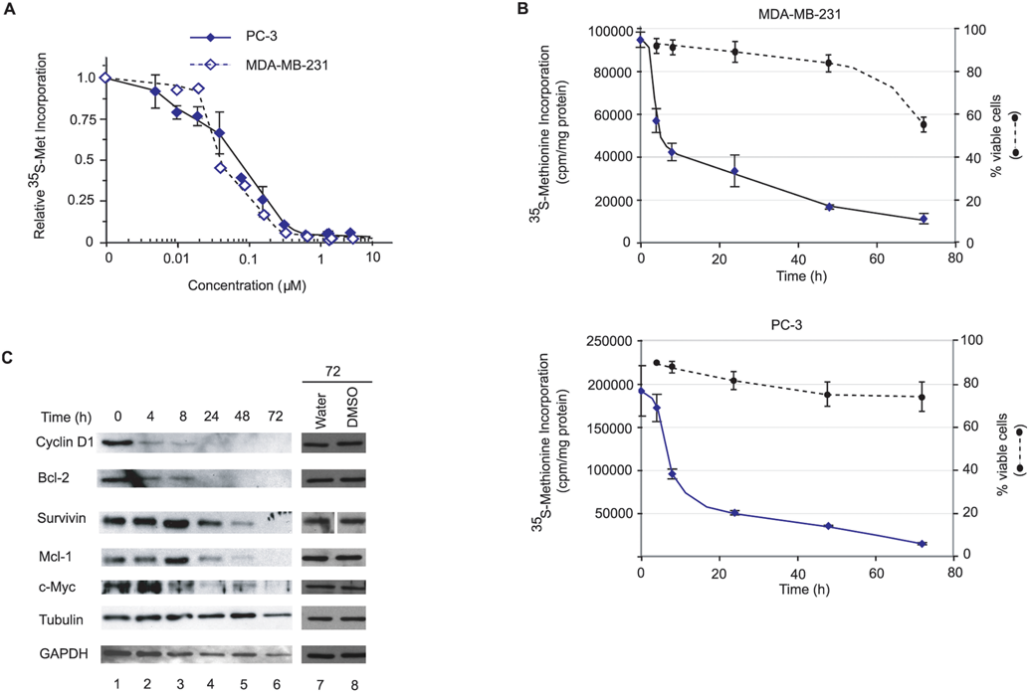
Silvestrol inhibits translation *in vivo* in MDA-MB-231 breast and PC-3 prostate cancer cells. A. Relative rate of ^35^S-Met incorporation in MDA-MB-231 breast and PC-3 prostate cancer cells as a function of silvestrol concentration. Cells were exposed to the indicated concentrations of silvestrol for 1 h in Met-free DMEM with 10% dialyzed FBS, during which the last 15 min, ^35^S-Met was added. Results are the average of duplicates with the error of the mean shown. Values are standardized against total protein content and plotted relative to DMSO controls, which were ∼100,000 and 400,000 cpm/µg for MDA-MB-231 and PC-3 cells, respectively. B. Kinetics of protein inhibition and cell death following exposure to silvestrol. Cells (60,000 per well in a 24-well plate) were exposed to 25 nM silvestrol for the indicated periods of time. One set of cells was used to quantitate protein synthesis following ^35^S-Met-labeling, TCA precipitation, and scintillation counting. A parallel set of dishes (200,000 cells per well in a 6-well plate) was used to measure the percentage of viable cells by Annexin V/P.I. staining followed by FACs analysis. These values were normalized to those obtained in the presence of vehicle (1% DMSO), which was set to 100%. C. Reduction in expression of eIF4E-responsive gene products in MDA-MB-231 cells exposed to silvestrol. Western blotting was used to evaluate protein levels from cell lysates harvested at the indicated time points. The panels for survivin (lanes 7 and 8) were analyzed on the same gel, but not on adjacent lanes.

Silvestrol inhibited protein synthesis in MDA-MB-231 breast and PC-3 prostate cancer cell lines with approximately the same IC_50_ (∼60 nM) following a 1 h exposure (Fig. 3A). We next determined the extent to which 25 nM silvestrol would affect protein synthesis rates in these cells. Translation rates were monitored as a function of time-post-exposure to silvestrol by labeling proteins with ^35^S-Met 15 min before harvest (Fig. 3B). A biphasic response was noted with a precipitous drop occurring over the first 8 h, followed by a slower reduction in translation occurring from 8–72 h (Fig. 3B). The reduction in translation rates was not a consequence of silvestrol-induced apoptosis since this was only observed for MDA-MB-231 cells during the last 24 h of the experiment (40% reduction in viability) (Fig. 3B). We noticed that during the first 8 h following inhibition of protein synthesis by silvestrol there was an ∼2 fold decrease in ^35^S-met incorporation into newly synthesized proteins for MDA-MB-231 (Fig. S3A: between 0–4 h for MDA-MB-231 cells - compare lane 2 to 1) and PC-3 (Fig. S3A: between 4–8 h for PC-3 cells: compare lane 3 to 2). By 24 h, we noted a reduction in the labeling of specific proteins (Fig. S3A: denoted by open boxes). In contrast, cycloheximide, an inhibitor of elongation, completely blocked protein synthesis and reduced ^35^S-Met incorporation equivalently in all proteins (data not shown).

These results indicate that treatment of cells with silvestrol might lead to mRNA discrimination during translation. To explore this, we generated reporter constructs in which a G-quadruplex had been engineered into the 5′ untranslated region of the mRNA 6 nucleotides from the site of transcription initiation (Fig. S4A, G-Q(+6)/RL). The G-quadruplex was modeled on one previously described to be present within the 5′ UTR of NRAS RNA and shown to mediate translational repression [23]. A control construct, [CAA]_10_/RL, generated a reporter transcript having a 5′ UTR consisting of [CAA] tracts and harboring minimal secondary structure with lowered eIF4F dependency [24]. As an internal control, we utilized a construct in which the HCV IRES was driving expression of firefly luciferase, an element known to recruit ribosomes in an eIF4F-independent manner [25]. Translation of G-Q(+6)/RL was more cap-dependent than [CAA]_10_/RL in Krebs extracts, as judged by inhibition of translation in the presence of m^7^GDP (Fig. S4B). In addition, G-Q(+6)/RL was more sensitive to reduced levels in eIF4A activity, as determined by inhibition of translation in the presence of the eIF4A inhibitor, hippuristanol [26]. In the presence of increasing concentrations of silvestrol, translation of G-Q(+6)/RL was inhibited to a greater degree than [CAA]_10_/RL mRNA (Fig. S4C), being consistent with inhibition of translation by silvestrol leading to mRNA discriminatory effects. This discriminatory effect was also observed with another flavagline, compound #7 (Fig. 1A), as well as on another set of reporter constructs in which the highly structured HIV TAR element was placed upstream of the chloramphenicol acetyl transferase (CAT) coding region (Fig. S5A). The PLTAR element is inhibitory to translation initiation and renders translation more sensitive to ionic concentrations [27] (Fig. S5B; compare lane 7 to 4). PLTAR CAT mRNA translation proved to be more sensitive to compound #7, than the CAT reporter (Fig. S5B, compare lanes 8–11), indicating that the results obtained with silvestrol are not compound- or reporter- specific (Fig. S4).

We next asked whether exposure of cells to silvestrol would affect the expression of malignancy-related proteins (Fig. 3C). We chose to evaluate the oncogenes cyclin D1 and c-myc and the anti-apoptotic proteins Bcl-2, survivin, and Mcl-1 since these have all been reported to be eIF4E-dependent. Levels of cyclin D1, Bcl-2 and c-myc diminished within the first 8 h of exposing MDA-MB-231 cells to silvestrol (Fig. 3C, compare lanes 1–3). Levels of survivin and Mcl-1 decreased substantially after 48 h, whereas tubulin and GAPDH expression was largely unaffected (Fig. 3C), consistent with the notion that silvestrol selectively affects production of growth-related proteins. Treatment of cells with vehicle for 72 hrs did not change levels of any of these proteins (Fig. 3C, compare lane 7 to 8). The levels of tubulin and GAPDH seen at 48–72 hrs in silvestrol treated cells are also similar to levels observed in untreated cells and thus cannot be attributed to selective enhancement of their synthesis by silvestrol (Fig. 3C, compare lanes 5 and 6 to 1). RT-PCR analysis of RNA isolated from unbound and polysome-bound fractions from DMSO- and silvestrol-treated MDA-MB-231 cells indicated that silvestrol leads to a redistribution of mRNA from polysomes into unbound fractions for these malignancy-related mRNAs (Fig. S3B). This is consistent with silvestrol inhibiting translation initiation of these transcripts.

We have previously shown that eIF2α is not phosphorylated in response to exposure of cells to silvestrol [4], and wanted to assess if inhibition of translation by silvestrol induced stress granule formation, a common event associated with inhibition of translation initiation. Arsenite, a known inducer of SGs caused relocalization of eIF4A and eIF4E into SGs, as determined by co-localization with the SG marker, G3BP (Fig. S6A, B). Exposure of HeLa cells to silvestrol also induced colocalization of eIF4A, eIF4E and G3BP into SGs (Fig. S6).

### Silvestrol reduces translation in normal mouse tissues without cytotoxicity

**Figure 4:**
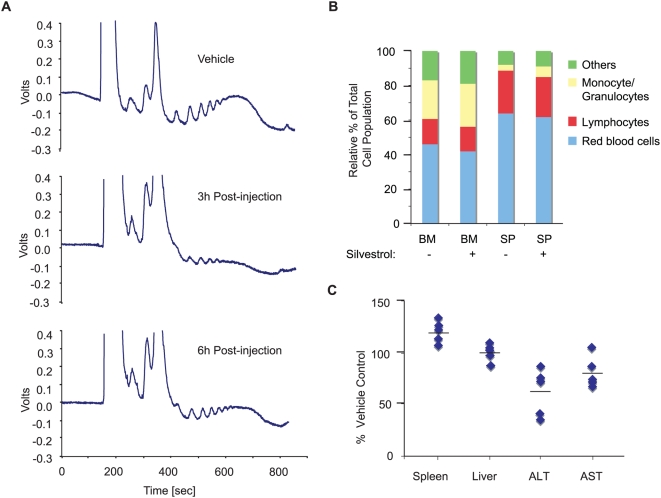
Long-term administration of silvestrol is well tolerated. A. Silvestrol causes a transient depression of protein synthesis in liver of mice receiving compound. Analysis of liver polysomes from mice injected two consecutive days with 0.2 mg/kg silvestrol and taken 3 or 6 h after the last injection. B. Following 8 consecutive daily administrations of silvestrol (0.2 mg/kg) into Balb/c male mice, bone marrow (BM) and spleen (SP) cell populations were quantitated by FACs analysis. The relative percentage (%) of each population is shown. C. Silvestrol does not alter spleen or liver weights or increase liver aminotransferase activity. Eight Balb/c mice were administered daily injections of vehicle or silvestrol for 8 days. Alanine (ALT) and aspartate (AST) aminotransferase levels, and spleen and liver weights were determined one day after the last injection. The bar represents the mean of the measurements set relative to levels obtained from control mice for each cohort.

The cap-dependent translation process is a target of silvestrol. Injection of silvestrol for two consecutive days into mice caused a reduction in liver protein synthesis, as assessed by polysome analysis in liver tissue isolated 3 and 6 h after the second injection (Fig. 4A). These results clearly indicate that silvestrol can inhibit protein synthesis *in vivo*. We then administered silvestrol to non-tumor bearing mice once a day for 8 consecutive days and assessed if there was an effect on red blood cells, lymphocytes, or monocyte/granulocyte counts from both bone marrow (BM) and spleen (SP) of control and silvestrol-treated mice (Fig. 4B). Vehicle- and silvestrol-treated animals displayed similar blood cell profiles (Fig. 4B). As well, there was no change in body weight of mice treated with silvestrol (Fig. S7) or any signs of illness or distress. Additionally, there was no appreciable change in liver or spleen weight (Fig. 4C). *In vivo* toxicity can be monitored by the appearance of enzymes in the serum such as ALT and AST. However, rather than an elevation of ALT or AST levels as expected for liver or muscle damage, we observed a slight reduction in AST levels and a 50% decrease in ALT levels, likely reflecting the inhibition of protein synthesis exerted *in vivo* by silvestrol (Fig. 4C). Collectively, these data indicate that silvestrol appears well tolerated in normal tissues.

### Silvestrol suppresses HUVEC cell growth

**Figure 5:**
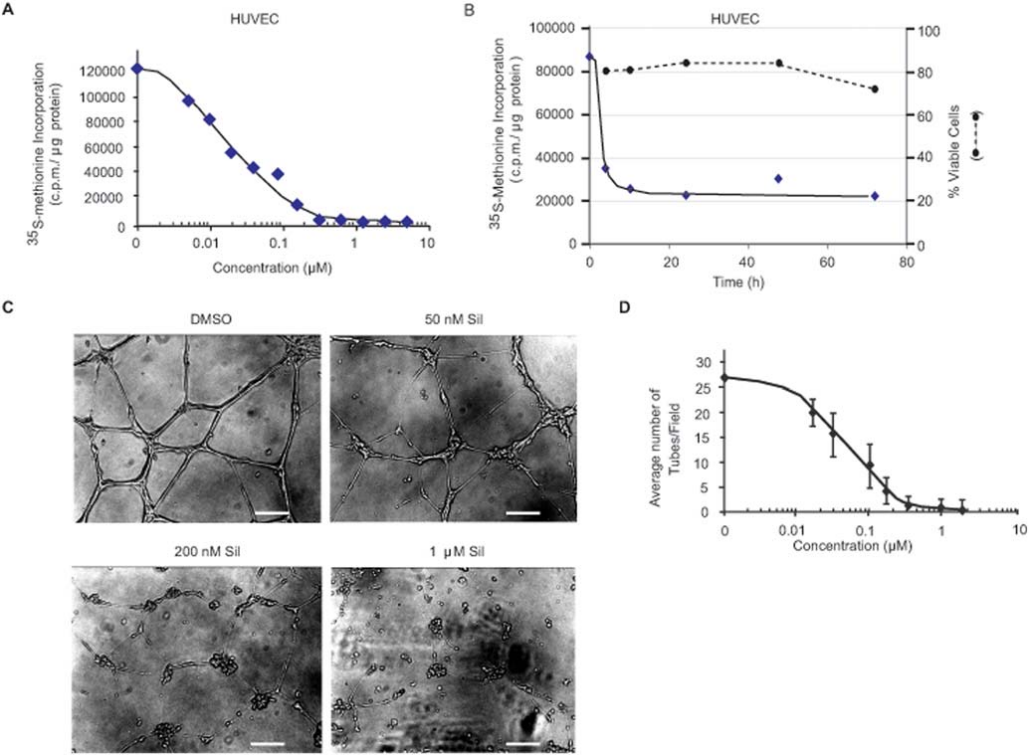
Silvestrol inhibits protein synthesis and suppresses endothelial cell tube formation. A. The relative rate of ^35^S-Met incorporation in HUVECs as a function of silvestrol concentration. Cells were exposed to the indicated concentrations of silvestrol for 1 h, and in the last 15 min, ^35^S-Met was added to the cells. Extracts were prepared and the amount of TCA-insoluble ^35^S-Met determined. Results are the average of duplicates with the error of the mean too small to be seen. Values are standardized against total protein content. B. Kinetics of protein inhibition versus cell death following exposure of HUVECs to silvestrol. HUVECs were exposed to 25 nM silvestrol for the indicated periods of time. One hour before the end of treatment, media was removed, cells washed with PBS and incubated for an additional hour with silvestrol in Met-free DMEM. For the last 15 min, cells were labeled with ^35^S-Met, followed by TCA precipitation and scintillation counting. A parallel set of dishes (200,000 cells/well in a 6-well plate) were used to measure the percentage of viable cells by Annexin V/P.I. staining and FACs analysis. These values were normalized to those obtained in the presence of 1% DMSO, which was set at 100%. These values are plotted on the right ordinate and as a dashed line. C. Disruption of tube formation by HUVECs in the presence of silvestrol. HUVECs were seeded on BD Matrigel™ Matrix basement membrane (BD Biosciences, Bedford MA) in 24-well plates in triplicate in the presence of increasing concentrations of silvestrol and 24 h later monitored for tube formation. Photographs were taken with a Nikon eclipse TE300 microscope. The bar at the bottom of each photograph corresponds to 50 µm D. Quantitation of tubules (chord-like structures) observed per field. A total of 15 different fields were used for each data point and the errors represent the standard deviation.

The potential consequences of silvestrol on angiogenesis have not been explored, so we evaluated if it could suppress the response of endothelial cells to angiogenic stimuli. Silvestrol inhibited protein synthesis in HUVEC cells (Figs. 5A, B) and did not induce apoptosis even when cells were exposed to compound for 72 h (Fig. 5B). We also evaluated the ability of silvestrol to prevent or delay formation of tube or chord-like structures by HUVECs. Though this *in vitro* model does not fully recapitulate the features of *in vivo* angiogenesis, it is none-the-less useful for evaluating the response of endothelial cells to angiogenic stimuli. Silvestrol prevented the ability of HUVECs cultured on Matrigel to form vessel-like structures (Fig. 5C). This was quantitated by counting the number of tubes per field and demonstrated that silvestrol caused a dose-dependent reduction in tube formation (Fig. 5D).

### Silvestrol suppresses xenograft tumor growth

**Figure 6:**
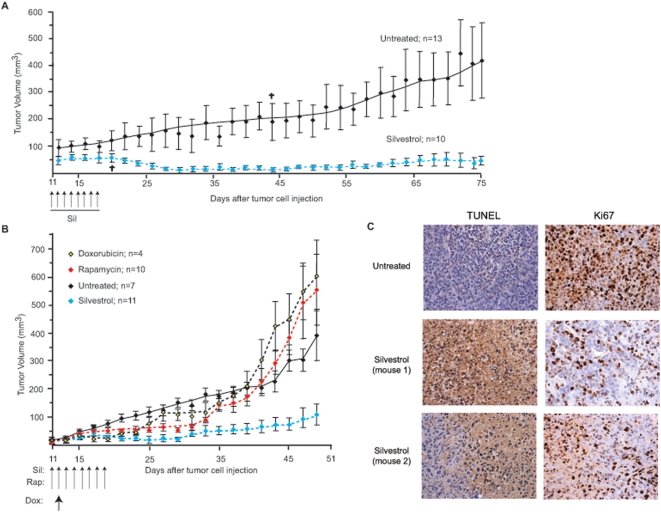
Silvestrol suppresses xenograft tumor growth. A. Response of nude mice bearing early passage human MDA-MB-231 breast cancer to silvestrol. Dosing schedule is indicated at the start of Day 11. Small crosses indicate loss of 2 animals during the course of the experiment. B. Response of nude mice bearing late passage human MDA-MB-231 breast cancer to silvestrol, doxorubicin, or rapamycin. Dosing schedule is indicated at the start of Day 11. C. Xenografts were evaluated by TUNEL and immunohistochemistry for Ki67. Representative data are shown for control-treated and silvestrol-treated mice.

Deregulated eIF4F activity has been postulated to contribute to the oncogenic process in breast cancers [28]. We therefore chose to evaluate the impact of silvestrol administration on tumor growth in MDA-MB-231 breast cancer xenografts (Fig. 6). At 11 days after implantation, silvestrol was administered intraperitonally at 0.5 mg/kg once per day for 8 consecutive days. Following treatment, mice were monitored for up to 2 months and we noted that silvestrol dramatically suppressed the growth of these tumors (Fig. 6A and B). In these experiments, we used two different passages of MDA-MB-231 cells, an early passage that appeared to be slower growing *in vivo* (Fig. 6A) and a late passage, faster growing, line (Fig. 6B). In contrast to silvestrol treatment, administration of doxorubicin or rapamycin did not inhibit tumor growth (Fig. 6B). However, synergy with doxorubicin was observed for both rapamycin and silvestrol *in vitro* when tested against MDA-MB-231 cells (Table 1). An additional xenograft model that we used was the PC-3 human prostate cancer model, since the AKT/mTOR signaling pathway is frequently upregulated in these cancers [29], [30]. Growth of PC-3 xenografts was also significantly reduced in mice dosed with 0.5 mg/kg silvestrol for 8 consecutive injections following appearance of the tumors 24 days after implantation (Fig. S8). This observation contrasts with the results obtained with Dox at 5 mg/kg, which showed little effect.

**Table 1:** Synergy of Doxorubicin with rapamycin and silvestrol on MDA-MB-231 cells.

Combination	Ratio	CI(ED50)	CI(ED75)	CI(ED90)	Average CI
Dox+Rap	1.47∶1	1.159	0.789	0.538	0.83
Dox+Silv (Exp #1)	2.94∶1	0.712	0.295	0.123	0.38
Dox+Silv (Exp #2)	11.79∶1	0.414	0.270	0.183	0.29
Dox+Silv (Exp #3)	0.18∶1	0.483	0.215	0.096	0.27

MDA-MB-231 tumors harvested at the end of the study were analyzed to determine the fraction of apoptotic and dividing cells (Fig. 6C). TUNEL staining showed a significant increase in silvestrol-treated tumor cells staining positive for apoptosis and proliferation of silvestrol-treated tumor cells was greatly reduced as judged by staining for Ki67 (Fig. 6C).

## Discussion

Deregulated translation initiation through elevated eIF4E levels and increased eIF4F activity has been repeatedly implicated in malignancy through the altered translation of pro-survival and pro-growth encoding mRNAs that contribute to oncogenesis [31], [32], angiogenesis [17], and chemoresistance [33]. Recently, integrated genomic analysis of human glioblastoma multiforme, pancreatic cancers, breast and colon cancers revealed that human cancers contain a large number of genetic alterations (e.g. an average of 63 genetic alterations for pancreatic cancers) that define a core set of cellular signaling pathways and processes that are altered in a large number of tumors (67–100% in pancreatic cancers) [34]. In the case of pancreatic cancers, these include K-ras signaling, invasion, Small GTPase-signaling, apoptosis, integrin signaling, DNA damage control, notch signaling, and JNK signaling – all pathways that converge on eIF4E [35]–[41]. Hence inhibiting the eIF4F checkpoint suppresses tumor growth through multiple mechanisms and provides a rationale for development of broad-acting therapeutics. We show here that targeting the eIF4A subunit of eIF4F in human cancer xenograft tissues is sufficient to reduce tumor cell proliferation.

Silvestrol was the most active cyclopenta[*b*]benzofurans inhibiting protein synthesis among those that we tested. Interestingly, this compound has the same core structure as compound #2, which did not inhibit cap-dependent protein synthesis to the same extent as other compounds tested - notably #6, 7 and 8. The acetate moiety on compound #7 seems to be an important contributor to increasing this compound's activity relative to compound #2. These results indicate that silvestrol's activity may be improved by incorporating this functional group. Clearly, the dioxane moiety of silvestrol is a major contributor to its activity. Cyclopent[*bc*]benzopyrans and benzo[*b*]oxepines did not show any inhibition of protein synthesis (Fig. S2) indicating that the core benzofuran ring system is essential for this property. Cyclopent[*bc*]benzopyrans and benzo[*b*]oxepines have shown no antiproliferative activity for cancer cell lines *in vitro*, consistent with the idea that inhibition of protein synthesis by cyclopenta[*b*]benzofurans is responsible for their anti-proliferative activity [42].

In the current study, we also provide mechanistic insight into the mode of action of silvestrol. We confirm that RNA binding of eIF4A_f_ is increased in the presence of silvestrol using an RNA filter binding assay and that this binding is inhibited by hippuristanol (Fig. 2A). The increased crosslinking of eIF4A_c_ to mRNA from RSW was shown to be cap-dependent (Fig. 2B). There are several possibilities to explain these results including stabilization of eIF4A_c_ in the eIF4F complex by silvestrol, increased recycling of eIF4A_f_ through the eIF4F complex during initiation, or altered conformation of eIF4A_c_ induced by silvestrol that favors crosslinking (e.g.-exposure of an amide residue). We find less eIF4A_c_ associated with the eIF4F complex when eIF4F is isolated by m^7^GTP-Sepharose affinity column from RSW or silvestrol-treated cells (Fig. 2C), consistent with the idea that silvestrol depletes the eIF4F complex of eIF4A. We find no increase in eIF4G associated with RNA-bound eIF4A indicating that the eIF4F complex is not sequestered to RNA by silvestrol (Fig. 2D).

We have previously reported that administration of silvestrol to mice bearing Eμ-myc derived lymphomas synergizes with standard-of-care agents, such as doxorubicin, to induce apoptosis *in vivo* [4]. However, as a single agent silvestrol was not effective at curtailing lymphoma development in this model [4]. In the current setting, silvestrol was effective as a single agent against two different human xenograft models, resulting in tumor growth arrest associated with massive apoptosis and halt of cellular proliferation (Fig. 6). The reasons for these differences are not immediately apparent, but may be related to the presence of an activated c-myc allele in the Eμ-myc model. Alternatively, silvestrol's anti-angiogenic effects (Fig. 5) may be quite important for its anti-proliferative activity against solid tumors, but less so towards lymphomas. Alternatively, it may be that the tumors used in the current study are more “addicted” to altered translation initiation rates (due to increased numbers of altered signaling pathways or processes) than the Eμ-myc/PTEN tumors previously used [4], and hence respond to single agent treatment. Interestingly, rapamycin showed no effect in the MDA-MB-231 xenograft model used here demonstrating the superiority of silvestrol in the two models tested (Fig. 6B). Although high concentrations of doxorubicin (10 mg/kg) were sufficient to achieve an anti-tumor response, the mice lost weight and did not thrive (R.C., data not shown). A single dose of doxorubicin at 5 mg/kg was not effective against MDA-MB-231 breast cancer cells *in vivo*, although rapamycin and silvestrol did show synergy with doxorubicin *in vitro* (Table 1).

Many key proteins involved in malignancy are translationally controlled, including the potent angiogenic factors VEGF and FGF-2, the oncogenes cyclin D1 and c-myc, the antiapoptotic proteins of the Bcl family, as well as the inhibitor of apoptosis protein survivin (refs. 30, 31; see ref. 1 for a more extensive list of translationally controlled proteins involved in malignancy). Indeed, modulation of eIF4E can directly affect the expression of many of these malignancy related proteins (reviewed in refs. 1, 30). One prediction is that silvestrol should preferentially inhibit the translation of mRNAs whose expression is more dependent on eIF4F for ribosome recruitment. Indeed, silvestrol (and Compound #7) showed a preference for affecting the translation of “weaker” mRNAs (Figs. 3C, S3B, S4 and S5). This discriminatory effect is consistent with silvestrol targeting the eIF4F complex and inhibiting ribosome recruitment. Our results do not exclude an additional effect of silvestrol on protein stability. The ability of silvestrol to induce formation of stress granules (Fig. S6) is expected for an inhibitor of translation initiation.

The inhibition of protein synthesis that we observed with silvestrol for MDA-MB-231 and PC-3 cells is very different than that noted with 4E-antisense oligonucleotides. We observed a biphasic response with a precipitous drop occurring over the first 8 h, followed by a slower reduction in translation occurring from 8–72 h (Fig. 3B). This result is in contrast to what has been reported for eIF4E antisense oligonucleotides, where no reduction in global protein synthesis was observed over the course of 72 h [17]. These differences may reflect the different requirements of translation initiation for eIF4A versus eIF4E, since ribosome recruitment to mRNAs containing unstructured 5′UTRs can be mediated by eIF4G/eIF4A in the absence of eIF4E [24]. Nonetheless, it does seem that inhibition of translation with silvestrol can result in mRNA discriminatory effects (Fig. S4).

Administration of silvestrol appeared well tolerated in non-tumor bearing mice without inducing appreciable toxicity (Fig. 4 and Fig. S7). In normal resting cells translation initiation may be very low as a large fraction of eIF4E may be complexed with the inhibitory 4E-BPs in the absence of Akt/mTOR pathway stimulation [28]. As well, in normal cells stimulated to proliferate, the spectrum of mRNA whose translation is elevated may differ significantly from those elevated in transformed cells. Consistent with this hypothesis, increased phosphorylation of 4E-BP (presumably leading to elevated eIF4F activity) in liver from mice administered the branched chain amino acid (which activates mTOR) stimulates ribosomal protein mRNA translation but not global rates of protein synthesis in liver [43]. Yet, altering eIF4E levels by transcriptional activation [44], knock down using shRNAs [44], or inhibiting mTOR activity [45] in transformed cells can affect mRNA-selective translation and global rates of protein synthesis (∼2-fold decrease). The alteration in global translation rates may be due to a larger pool of mRNAs that are discriminated by eIF4E in tumor cells. Using anti-sense oligonucleotides to knockdown expression of eIF4E in the mouse is also well tolerated [17]. Our results support the idea that curtailing translation initiation by modulating eIF4A activity is a promising anti-cancer therapeutic approach that is well tolerated.

## Materials and Methods

### Ethics Statement

All animal studies were approved by the McGill University Faculty of Medicine Animal Care Committee

### General Reagents

Silvestrol was resuspended in DMSO and stored at −70°C. Doxorubicin (Sigma) was dissolved in water and stored at 4°C. Rapamycin (LC Laboratories, Woburn, MA) was resuspended in 100% ethanol and stored at −70°C.

### Cell culture

Malignant metastatic human mammary epithelial MDA-MB-231 cells were obtained from the American Type Culture Collection (ATCC, Rockville, MD) and grown as monolayers in L15 medium (Invitrogen) supplemented with 10% fetal bovine serum and 100 U/ml penicillin/streptomycin. PC-3 cells were obtained from the American Type Culture Collection (ATCC, Rockville, MD) and grown as monolayers in F12-K medium (Invitrogen) supplemented with 10% fetal bovine serum and 100 U/ml penicillin/streptomycin. HUVEC cells were obtained from Lonza (Walkersville, MD) and propagated in EBM-2 medium supplemented with EGM-2.

### Recombinant DNA constructs and in vitro translations

The bicistronic reporter, FF/HCV/Ren, has been previously described [46]. For generating reporter constructs containing a G-quadruplex or a [CAA] tract for *in vitro* translation assays, the plasmid phRL-null was used (Promega). This plasmid was linearized with *Nhe*I/*Nco*I and gel purified. Two sets of annealed oligonucleotides were inserted into these sites. One set [sense: ^5′^CTAG[CAA]_10_C^3′^ and antisense: ^5′^CATGG[TTG]_10_^3′^ ] generated [CAA]_10_/RL, whereas Set II [sense: ^5′^CTAGGGGAGGGGCGGGTCTGGG[CAA]_4_C^3′^ and antisense: ^5′^CATG[GTT]_4_CCCAGACCCGCCCCTCCC^3′^ ] generated G-Q(+6)/RL. Plasmid G-Q(+6)/RL contains the G quadruplex from NRAS positioned six nucleotides downstream from the T7 RNA polymerase transcription start site [23]. Plasmids encoding CAT or PLTARCAT have been previously described [47].

For *in vitro* translations, constructs G-Q (+6)/RL and [CAA]_10_/RL were linearized with *Xba*I followed by *in vitro* transciption with T7 RNA polymerase. Plasmid pSP72/HCV/Luc A+ (a gift from Dr. N. Sonenberg [McGill University]) was linearized with *Bam*HI and subsequently *in vitro* transcribed with T7 RNA polymerase. *In vitro* translations where performed with 0.2 ng/µl *in vitro* transcribed RNAs in micrococcal nuclease treated Krebs extract as described previously [46] with the indicated concentrations of silvestrol for 1 h at 30°C. FF and Ren luc activity (RLU) were measured on a Berthold Lumat LB 9507 luminometer. *In vitro* translations of FF-HCV-Ren in RRL (Promega) were performed according to the manufacturer's instructions.

### Filter Binding and Crosslinking Assays

Filter binding and chemical crosslinking assays were performed as previously described [22], [48]. For crosslinking assays, a 30 µL reaction containing 10 µL ribosomal salt wash (RSW) (1.2 µg/µL) was incubated under standard conditions (25 mM Hepes [pH 7.5], 70 µM GTP, 11 µM of each of the amino acids, 2 mM DTT, 60 µM PMSF and 0.5 mM Mg(OAc)_2_) with 0.9 mM ATP (unless indicated otherwise) in the presence of oxidized ^32^P-labeled CAT RNA (50,000 cpm). Reactions were incubated for 10 min at 30°C and then crosslinked using 20 mM NaBH_3_CN overnight at 4°C. After treatment with RNAse A, proteins were separated by 10% SDS-PAGE and visualized by autoradiography (Kodak X-Omat).

### ^35^S-methionine labeling and Western blotting

Malignant metastatic human mammary epithelial MDA-MB-231 cells were obtained from the American Type Culture Collection (ATCC, Rockville, MD) and grown as monolayers in L15 medium (Invitrogen) supplemented with 10% fetal bovine serum and 100 U/ml penicillin/streptomycin. PC-3 cells were obtained from the American Type Culture Collection (ATCC, Rockville, MD) and grown as monolayers in F12-K medium (Invitrogen) supplemented with 10% fetal bovine serum and 100 U/ml penicillin/streptomycin. HUVEC cells were obtained from Lonza (Walkersville, MD) and propagated in EBM-2 medium supplemented with EGM-2.

To measure the rate of ^35^S-Met incorporation into protein, 60,000 cells/well were seeded in a 24-well plate. The following day, the medium was removed, cells washed with PBS and exposed to silvestrol at the indicated concentrations in methionine-free DMEM supplemented with 10% dialyzed serum for 1 hr. For the last 15 min, cells were labeled with ^35^S-methionine. Medium was removed, cells washed in PBS and lyzed in RIPA buffer (20 mM Tris [pH 7.5], 100 mM NaCl, 1 mM EDTA, 1 mM EGTA, 0.1% NP-40, 0.5% sodium desoxycholate, 0.1% SDS, 20 mM ß-glycerophosphate, 10 mM NaF, 1 mM PMSF, 4 µg/ml aprotinin, 2 µg/ml leupeptin, 2 µg/ml pepstatin) for 20 min with shaking at 4°C. The protein was TCA precipitated and the radioactivity quantitated by scintillation counting. Protein content in the cell lysates was measured using the Bio-Rad D_C_ ProteinAssay (Bio-Rad Laboratories) and used to standardize the counts obtained by TCA precipitation.

To visualize ^35^S-methionine labeled proteins, equal amounts of extracts were resolved on 10% SDS-polyacrlamide gels, stained with Coomassie Blue to verify equivalent loading, treated with En^3^Hance, dried and exposed to X-OMAT X-ray film (Kodak).

To monitor cellular viability, 200,000 cells/well were seeded in a 6-well plate and treated with 25 nM silvestrol for the indicated times. At the end of the treatment, the cell media was collected, cells were washed with 1 ml PBS and trypsinized with 200 µl trypsin. Cells were collected and pooled with the initial media and PBS wash. Samples were spun at 4°C for 2 min at 2000 rpm in a Sorval LegendRT table centrifuge. The pellet was resuspended in 2 ml cold PBS followed by another 2 min spin at 2000 rpm. The pellet was resuspended in 100 µl Annexin V binding buffer (10 mM HEPES [pH 7.4], 140 mM NaCl, 2.5 mM CaCl_2_) followed by the addition of propidium iodide to a final concentration of 5 µg/ml. After addition of 5 µl Annexin V-FITC (BD- Biosciences), samples were incubated for 15 min at RT in the dark followed by the addition of 400 µl of Annexin V binding buffer. FACS analyses were performed using a FACScan instrument from BD Biosciences and CELLQUEST software.

For Western blot analysis, cells were grown in 6-well plates, washed with PBS, harvested with a rubber policeman, and collected by brief centrifugation. Cell pellets were lyzed in RIPA buffer and separated on a 10% SDS-polyacrylamide gel followed by transfer to a PVDF membrane (Millipore). Primary Antibodies used were anti-Cyclin D1 (Cell Signaling, Danvers, MA), anti-Bcl-2 (Cell Signaling, Danvers, MA), anti-c-myc (Santa Cruz, Santa Cruz, CA), anti-Mcl-1 (Rockland, Gilbertsville, PA), anti-Survivin (Novus, Burlington, ON), and anti-tubulin (Sigma-Aldrich, Oakville, ON) and anti-GAPDH (Abcam, Cambridge, MA). Secondary antibodies were from Jackson Immuno Research (Burlington, ON).

### Monitoring Stress Granule Formation

Anti-eIF4A and anti-G3BP antibodies have been previously described [49], [50]. Anti-eIF4E antibody was a gift of S. Kimball and has been described previously [51]. Cells were processed for immunofluorescence as previously described [50]. Essentially, cells were fixed in 3% paraformaldehyde and permeabilised with 0.1% Triton-X100/PBS. Slides were incubated with primary antibodies diluted in 0.1% normal goat serum for 1 h at RT. Following washing, the slides were incubated with goat anti-mouse/rabbit IgG (H+L) secondary antibodies coupled to goat Alexa Fluor 488/594. Fluorescence microscopy was performed using a Zeiss Axiovision 3.1 microscope equipped with Axiocam HR (Zeiss) digital camera. Images were compiled using Adobe Photoshop software.

### Isolation of eIF4F from RSW and MDA-MB-231 cell extracts

RSW was incubated in the presence of 0.5% of DMSO or 50 µM silvestrol for 1 h at 30°C, followed by the addition of 50 µL of 50% m^7^GTP-Sepharose beads (G.E. Healthcare). The reactions were incubated for 2 h end-over-end at 4°C, after which the beads were washed three times with 240 µL of LCB (20 mM HEPES [7.5], 100 mM KCl, 0.2 mM EDTA), and twice with 240 µL of LCB+ 200 µM GDP. Proteins were eluted with 120 µl of LCB+ 200 µM m^7^GTP for 10 min on ice.

For eIF4F pulldown experiments from cell extracts, 8×10^6^ MDA-MB-231 cells were seeded into 15 cm^2^ dishes and the next day treated with 25 nM silvestrol for 4 h. Cells were washed in cold PBS, scraped with a rubber policeman and spun down for 5 min at 2500 rpm. Cell pellets were resuspended in lysis buffer (20 mM Tris [7.5], 100 mM KCl, 1 mM DTT, 1 mM EDTA, 0.2% Tween20, 20 mM ß-glycerophosphate, 10 mM NaF, 1 mM PMSF, 4 µg/ml aprotinin, 2 µg/ml leupeptin, 2 µg/ml pepstatin) and immediately put on dry ice. Following 3 freeze-thaw cycles, extracts were centrifuged for 10 min at 14,000×g to remove cell debris. Pulldowns were performed with 1 mg of total protein extract and 50 µl of 50% m^7^GTP-Sepharose beads (GE Healthcare) for 2 h end-over-end at 4°C. Beads were washed 3 times with lysis buffer, once with LCB+ 500 µM GDP and proteins eluted with LCB+ 500 µM m^7^GTP for 10 min on ice. m^7^GTP elutions were analyzed on a 10% polyacrylamide gel, followed by transfer to a PVDF membrane.

For poly (rG) pulldown experiments, 500 µL of Krebs-2 extract was incubated with 0.5% DMSO, 10 µM pateamine or 50 µM silvestrol for 10 min at 4°C, followed by the addition of 50 µl of 50% Poly(rG) agarose (Sigma). Pulldowns were incubated for 1 h at 4°C. After incubation, the beads were washed twice with 10 volumes wash buffer (20 mM HEPES [7.5], 250 mM KOAc, 0.1% NP-40, 1 mM DTT). SDS elutions were resolved on a 10% SDS-polyacrylamide gel and transferred to PDVF membranes (Millipore). Antibodies used were anti-eIF4A [10] and anti-eIF4GI (Bethyl, Montgomery, TX), respectively. Secondary antibodies were from Jackson Immuno Research (Burlington, ON).

### Drug Synergy Assessment

MDA-MB-231 cells were seeded at 10,000 cells/well in a 96- well plate. The cells were allowed to adhere to the bottom of the plates for 24 h in complete media and then exposed to drug or vehicle in fresh media for the indicated periods of time. At the end of treatment, cells were washed with PBS followed by addition of 200 µl PBS to each well. Cell proliferation was monitored using the sulforhodamine B (SRB) assay [52]. Drug interaction was assessed by the combination index method of Chou and Talalay using CalcuSyn software (BioSoft, Cambridge, UK) [53].

### Animal Studies

For xenograft models, 5×10^6^ MDA-MB-231 or PC-3 cells were injected with matrigel sub-cutaneously into the right flank of 4–6 weeks old female Balb/c nu/nu mice. Tumor growth was monitored every day using calipers. Treatments were started when tumors had reached 25–50 mm^3^. Nude mice bearing tumors were dosed I.P. with vehicle (5.2% PEG400/5.2% Tween80), silvestrol (0.5 mg/kg), doxorubicin (5 mg/kg), or rapamycin (4 mg/kg). Tumor growth was then monitored for the remainder of the experiment and no further injections were made. For fixation, tumors were fixed in 10% formalin and embedded in paraffin for further analysis. TUNEL staining was performed using the In Situ Cell Death Detection Kit, POD (Roche) following the manufacturer's protocol. For Ki67 staining, the rabbit monoclonal Ki67 (Clone SP6) antibody was purchased from Thermo Scientific. Antigen retrieval was performed by boiling samples for 15 min in 10 mM Citrate buffer [pH 6.5]. Sections were incubated with primary antibody for 1 h at RT. The Ultravision Detection System Anti Rabbit, HRP,DAB (Thermo Scientific) was used according to the manufacturer's instructions.

To assess cytotoxicity of silvestrol, six male wt Balb/c mice, 8 weeks old, were treated with vehicle (5.2% PEG 400/5.2% Tween-80) or 0.2 mg/kg silvestrol for 8 consecutive days. Fresh cell suspensions of bone marrow (BM) and spleen (SP) were prepared in PBS+ 2% FBS. Nonspecific binding was blocked by incubation of the samples with purified anti-CD16/CD32 antibody (clone: 2.4G2; BD Biosciences) for 5 mins on ice before labeling of the cells with a combination of fluorochrome conjugated substrate specific antibodies. Antibodies used to identify monocytes and granulocytes were: Ly-6G/Ly-6C (Gr-1) PECy7 (clone RB6-8C5; Biolegend) and CD11b PE (clone M1/70; BD Biosciences). Antibodies used to identify T and B lymphocytes were: CD4 FITC (clone RM4-5; BD Biosciences), CD8 CyChrome (clone 53-6.7; BD Biosciences) and CD45R/B220 APC (clone RA3-6B2; BD Biosciences). Red blood cells were identified with the antibody Ter119 Biotin (clone ter119; Biolegend) followed by a streptavidin Pacific blue conjugated antibody (Invitrogen/Molecular Probes). Incubation was performed in the dark on ice for 30 min before data acquistion and analysis was conducted on a FACSAria machine (BD Biosciences) by using CELLQUEST (BD Biosciences) or FlowJo (Treestar) softwares.

Polysome profiling analysis on liver extracts was performed on male wt Balb/c mice, 6–8 weeks old. Mice were injected with vehicle (5.2% PEG 400/5.2% Tween-80) or 0.2 mg/kg silvestrol for 2 days. Three and six hours after the second injection, animals were sacrificed, and the livers excised and washed in cold PBS containing 100 µg/ml cycloheximide. The dissected livers were homogenized in a Eurostar Power-b homogenizer (IKA Liver Labortechnik, Staufen, Germany) in 3 volumes of lysis buffer (40 mM HEPES [7.5], 100 mM KCl, 5 mM MgCl_2_, 100 µg/ml cycloheximide). Homogenates were then centrifuged for 10 min at 1,200×g at 4°C. The supernatant was transferred into a new tube and to 150 µl of extract, 300 µl of lysis buffer containing 0.5% Triton X-100 and 0.5% sodium deoxycholate were added and the sample centrifuged briefly. Samples were loaded on 10–50% sucrose gradients and centrifuged in an SW40 rotor at 35,000 rpm for 2 h. Gradients were analyzed by piercing the bottom of the tubes with a Brandel tube piercer and passing 60% sucrose through the bottom. Recording of the data was performed using InstaCal Version 5.70 and TracerDaq Version 1.9.0.0 (Measurement Computing Corporation, Norton, MA).

### NOTE ADDED IN PROOF

While this manuscript was under review, Lucas *et al.* (Lucas, DM, Edwards, RB, Lozanski, G, *et al.* The novel plant-derived agent silvestrol has B-cell selective activity in chronic lymphocytic leukemia and acute lymphoblastic leukemia in vitro and in vivo. Blood, 2009; [Epub ahead of print]) also showed reduction of Mcl-1 protein following silvestrol treatment of acute lymphoblastic and chronic lymphocytic leukemias.

## Supporting Information

Figure S1 Relative potency of cyclopenta b benzofurans on cap- and HCV-mediated translation initiation. A. Schematic diagram of FF/HCV/Ren mRNA reporter used to program *in vitro* translation extracts. B. Krebs-2 translation extracts were programmed with FF/HCV/Ren mRNA and vehicle (MeOH) or 50 µM compound. A representative autoradiograph of an *in vitro* translations performed in the presence of [^35^S]-methionine is shown. Following separation of protein products on 10% SDS-polyacrylamide gels, the gels were treated with EN^3^Hance, dried, and subjected to autoradiography. The position of migration of FF and Ren luciferase proteins is indicated to the right.
(1.55 MB EPS)

Figure S2 Translation properties of cyclopenta bc benzopyrans and benzo b oxepines. A. Chemical structure of cyclopenta bc benzopyrans and benzo b oxepines tested in this study. B. Effect of compounds on cap- and HCV-mediated translation initiation. Krebs-2 translation extracts were programmed with FF/HCV/Ren mRNA and vehicle (MeOH) or 50 µM compound added. A representative autoradiograph of an *in vitro* translation reaction performed in Krebs-2 extracts with [^35^S]-methionine is shown. Following separation of protein products by SDS-PAGE, the gels were treated with EN^3^Hance, dried, and subjected to autoradiography. The position of migration of FF and Ren luciferase proteins is indicated to the right.
(6.70 MB EPS)

Figure S3 Effect of silvestrol exposure on global protein synthesis in MDA-MB-231 and PC-3 cells. A. Cells were exposed to 25 nM silvestrol for the indicated time points and radiolabeled with ^35^S-Met for the last 15 min of incubation. Cell extracts were prepared and equivalent protein amounts were loaded on 10% SDS-polyacrylamide gels. After electrophoresis, gels were stained with Coomassie blue to verify equal loading, treated with En^3^Hance, dried, and exposed to X-OMAT X-ray film (Kodak). Open squares identified some proteins whose abundance was reduced after 24 h. B. Six million cells (MDA-MB-231) were seeded in 15 cm^2^ dishes 24 h before treatment with 25 nM silvestrol or vehicle (DMSO) for 1 h. Cells were harvested by scraping with a rubber policeman in cold PBS containing 100 µg/ml cycloheximide and centrifuged for 10 min at 2000×g at 4°C. Pellets were resuspended in 425 µl hypotonic lysis buffer (5 mM Tris _7.5_, 2.5 mM MgCl_2_, 1.5 mM KCl) followed by addition of 5 µl 10 µg/ml cycloheximide, 1 µM DTT, 0.5% Triton X-100 and 0.5% sodium deoxycholate. Samples were loaded on 10–50% sucrose gradients and centrifuged in an SW40 rotor at 35,000 rpm for 2 h. Gradients were analyzed by piercing the bottom of the tubes with a Brandel tube piercer and passing 60% sucrose through the bottom. Fractions were collected from the gradients and monitored with an ISCO UA-6 UV detector (left panel). Fractions were separated into unbound and polysome (Poly)-bound regions. RNA was isolated using Trizol according to the manufacturer's instructions (Invitrogen). One-twentieth of the pooled RNA fractions was reverse transcribed using SuperScript™ II (Invitrogen) primed with oligo(dT)_12–18_. PCRs were performed with undiluted, 1∶10 or 1∶100 diluted cDNA samples. Primers used were hCyclin D1 [5′ CTCCTCTCCGGAGCATTTTGAT 3′ and 5′ CACCGCTCAGGGTTATGCAAAT 3′], hBcl-2 [5′ TGATGGGATCGTTGCC 3′ and 5′ CGCGGAACACTTGATT 3′], hSurvivin [5′ GGCCCAGTGTTTCTTCTGCTT 3′ and 5′ TTGACAGAAAGGAAAGCGCAAC 3′], hMcl-1 [5′ TTCAGCGACGGCGTAACAAACT 3′ and 5′ CCCATCCCAGCCTCTTTGTTTA 3′], and hc-Myc [5′ AAGAAATTCGAGCTGCTGCCCA 3′ and 5′ AACTCTGGTTCACCATGTCTCC 3′]. Annealing temperatures for hBcl-2, hSurvivin and hMcl-1 were 55°C and annealing temperatures for hCyclin D1 and hc-Myc were 59°C and 63°C, respectively. PCRs were optimized to detect the exponential phase of amplification and analyzed on 1.5% agarose gels (right panel).
(10.21 MB EPS)

Figure S4 Silvestrol inhibition of translation leads to mRNA discrimination. A. Schematic diagram of constructs used in this study. The renilla and firefly luciferase ORFs are represented by grey and blackened boxes, respectively. B. *In vitro* translation of mRNAs performed in Krebs extracts programmed with 0.2 µg/ml HCV/FF and 0.2 µg/ml of either [CAA]_10_/RL or G-Q(+6)/RL mRNA. The concentrations of hippuristanol, m^7^GDP, or GDP present in the reactions are indicated. Luciferase values were read on a Berthold Lumat LB 9507 luminometer. Renilla luciferase values were normalized to the firefly luciferase values and calculated relative to the vehicle control (set at 1). The values represent the average of 2-4 reactions with the error of the mean shown. Renilla luciferase values from the vehicle control were ∼20,000 and 150,000 RLU for translations programmed with G-Q(+6)/RL and [CAA]_10_/RL, respectively. Firefly luciferase values for vehicle-containing translations programmed with HCV/FF were ∼250,000 RLU. C. *In vitro* translation of mRNAs in the presence of increasing concentrations of silvestrol. Translations were performed in Krebs extracts programmed with 0.2 µg/ml HCV/FF and 0.2 µg/ml of either [CAA]_10_/RL or G-Q(+6)/RL mRNA. The concentrations of silvestrol present in the translation reactions are indicated. Luciferase values were read on a Berthold Lumat LB 9507 luminometer. Renilla luciferase values were normalized to the firefly luciferase values and calculated relative to the vehicle control (which was set at 1). The values represent the average of 2–4 reactions, with the error of the mean shown. The average firefly [HCV/FF] and renilla [G-Q(+6)/RL] values obtained with the vehicle controls were ∼320,000 and ∼16,000 RLU, respectively. Average values for renilla obtained from translation of [CAA]_10_/RL mRNA were ∼100,000 RLU.
(1.44 MB EPS)

Figure S5 mRNA discrimination by flavaglines is neither silvestrol- nor reporter- specific. A. Predicted secondary structure of PLTAR. B. Translation products of the indicated mRNAs from Krebs extracts containing KOAc concentrations of 75 mM (lanes 4 and 8), 100 mM (lanes 5 and 9), 125 mM (lanes 6 and 10), and 150 mM (lanes 7 and 11). The presence of vehicle (lanes 4–7) or 1 µM Compound 7 (lanes 8–11) is indicated. Fluorographs of the dried gels are presented.
(5.02 MB EPS)

Figure S6 Silvestrol induces the formation of SGs in HeLa cells. A. Distribution of eIF4A in HeLa cells upon exposure to arsenite or silvestrol. Cells were exposed to DMSO (0.5%) (top panel), arsenite (0.5 mM for 1 h) (middle panel), or silvestrol (5 µM for 1 h) (bottom panel), fixed and stained for eIF4A and G3BP. B. Distribution of eIF4E in HeLa cells upon exposure to arsenite or silvestrol.
(3.62 MB EPS)

Figure S7 Silvestrol does not cause weight loss over time. Eight Balb/c mice were administered silvestrol (0.2 mg/kg) on a daily basis for 8 days and their weights monitored at the indicated times.
(0.65 MB EPS)

Figure S8 Silvestrol suppresses tumor growth in a PC-3 prostate cancer xenograft model. Nude mice bearing human PC-3 prostate cancer (∼35 mm^3^) were dosed I.P. with vehicle (5.2% PEG400/5.2% Tween80), silvestrol (0.5 mg/kg), doxorubicin (5 mg/kg), or rapamycin (4 mg/kg). Dosing schedule is indicated at the start of Day 24.
(1.06 MB EPS)
